# Attribution of Health Hazards to Sources of Air Pollution Based on Networks of Sensors and Emission Inventories

**DOI:** 10.3390/s26010132

**Published:** 2025-12-24

**Authors:** Piotr Kleczkowski, Aleksandra Król-Nowak

**Affiliations:** Department of Mechanics and Vibroacoustics, AGH University of Krakow, 30-059 Krakow, Poland; akrol@agh.edu.pl

**Keywords:** health hazard, key air pollutants, sensor networks, air pollution sources, source apportionment

## Abstract

**Highlights:**

**What are the main findings?**

- Contributions of air pollution sources to health hazards can be estimated from databases of emissions.
- A simple analysis scheme based on these data confirmed that domestic heating was a prominent health hazard in Poland, which was also indicated by other analyses.

**What is the implication of the main finding?**

- Policymakers can be easily provided with adequate information on comparing hazards from particular sources of air pollution.

**Abstract:**

Air pollution is monitored worldwide through networks of sensors. They provide information on local air pollution, which also provides a basis for a multitude of research. To reduce health hazards caused by air pollution, the concentrations of pollutants as measured by sensors need to be apportioned to particular sources. Although several methods to achieve this have been developed, only a few works on the contributions of pollution sources to health hazards are available in the literature. In this work, a simple scheme is proposed to compare health hazards from each of the main sources of air pollution in a given country, region, or area. The comparison involves the main air pollutants of PM_2.5_, NO_2_, and O_3_ for chronic exposures and PM_2.5_, NO_2_, O_3_, and SO_2_ for acute exposures. The actual health hazard from each substance is determined from concentrations measured by sensors and the concentration–response functions found in the literature. The apportionment of substances to sources is based on emission inventories, thus avoiding costly methods of source apportionment. This method has been applied to the entire country, i.e., Poland, yielding the average proportion of health hazards from particular sources. The example demonstrates the flexibility and ease of application of the scheme. Uncertainties in the results were subjected to discussion. The key advantage of the scheme lies in its ability to provide an indication of the most harmful sources of pollution, thus highlighting efficient interventions.

## 1. Introduction

According to the World Health Organization (WHO), 99% of the global population breathe air containing pollutants that exceed WHO guideline limits [1]. In North America and in the European Union, emissions of air pollutants have declined over recent decades through appropriate policies and interventions, and an improvement in air quality has also been observed in China [2]. However, in many low- and middle-income countries, little progress has been made, and, in some cases, there has even been an increase in ambient air pollution levels [2] due to an increasing number of vehicles and the intensity of wildfires.

Air pollution is monitored worldwide by networks of sensors. Many countries have a monitoring system consisting of ambient air pollution measurement stations where accurate, professional instruments or sensors are installed to detect a number of pollutants [3,4]. The stations conform to strict rules and are operated by governmental agencies. These professional systems are complemented by less formalized networks of low-cost sensors [5,6,7,8]. The latter provides measurements of lower accuracy, but this is offset by more dense grids of measurement points capable of providing more detailed information on the spatial distribution of pollutants. Current information from sensors is used by local residents and administration, while past data serve as a basis for a wide range of research.

Policies for the reduction in pollution levels consist of changes in technology and practices, both of which are costly and may be considered inconvenient by individuals, businesses, or public administration. Hence, it is essential that the interventions are carefully chosen to be efficient. Information from networks of sensors, even if processed to obtain the spatiotemporal data needed, is not helpful in policy planning. The interventions can only be implemented on sources of pollution, not on substances. Therefore, contributions from sources to total measured pollution in a given location, area, region or an entire country must be recognized. Methods for source apportionment have been developed and can be grouped into two families: receptor models (based on chemical mass balance) and source-oriented models, based on emission inventories and modeling dispersion [9,10]. Accurate apportionment is difficult as pollutants undergo complex physical and chemical processes during the transport from the source to the receptor (measurement spot) in the atmosphere.

A level of pollution, i.e., a measured concentration of a pollutant (either local or averaged in space and/or time) is a measure of human exposure to that pollutant. Since one is able to apportion pollution to sources, the contributions of particular sources in the total exposure to each of the pollutants may be determined. This is the basic information that policymakers have used so far. In order to make air pollution policies more efficient, the decision makers need more information: Which pollutants are more harmful to human health than the others? Accounting for this factor will help allocate resources and efforts to particular sources of pollution, to provide maximal health benefits.

Quantification of the relationship between a pollutant and its health outcomes is a wide and difficult task involving toxicology and epidemiological studies. It requires narrowing of the objective, e.g., what type of outcome one is looking for, specific health effects or all-cause morbidity or mortality, chronic (long-term, associated with exposures measured in years) effects or acute (short-term, measured in days or weeks) effects. The subject has a wide coverage in the literature and databases [11,12] (all health hazards) and [2,13,14] (air pollutants with the strongest evidence for health concern).

The literature on the subject includes works analyzing the general issue and methodology of the dependencies between sources of air pollution and health outcomes [15,16,17,18,19,20,21]. These works provide general background and analyze various approaches, but they include no attempts to quantitatively evaluate contributions of sources to health hazards. Few papers presenting such an attempt are available.

Belis and Dingenen [10] investigated the forecasted effects of air pollution policies after the year 2020 on exposure to two pollutants: ozone (O_3_) and particulate matter (PM_2.5_: 2.5 μm fraction), and their associated mortality. They performed source apportionment with the use of a dispersion model and determined health impacts from a number of source types, estimating mortality according to the approach presented in an appendix to [11], which is based on results from epidemiological studies. They compared two macroregions: UNECE (United Nations Economic Commission for Europe)—56 countries of Europe, North America, West, North and Central Asia, with the Rest of the World (ROW) and quantified health hazards in mortality rate attributable to each source of air pollution. They found agriculture as the main contributor to mortality from PM_2.5_, followed by industry and domestic and commercial combustion (UNECE countries in 2020). They assessed the total mortality rate from PM_2.5_ (both natural and anthropogenic sources) in UNECE countries at 444,000 a year. Total mortality rate from O_3_ in UNECE in 2020 was estimated at 65,000. They did not aggregate mortality estimates from PM_2.5_ and O_3_ from the same sources.

Kim et al. [22] analyzed health hazards from around 60 hazardous air pollutants (HAPs) in two Korean cities: Seoul and Incheon. HAPs are toxic chemical compounds known to cause cancer and other serious health conditions. U.S. Environmental Protection Agency (EPA) currently lists 188 HAPs. Most of them are not regulated, as they are not commonly present in the atmosphere. HAPs are different than main air pollutants (see Section 2.2), although many of them are contained in PM. One of the objectives of Kim et al. was to estimate source-specific risks brought about by HAPs. Only a few HAPs are measured by public networks of sensors, so they performed their own measurements followed by source apportionment. They estimated health hazards from unit risks as given in EPA [12] and WHO databases and reference concentrations for each HAP. Finally, they expressed the contributions (as percentages) from all nine chosen groups of sources to total health hazards (cancer and non-cancer) from HAPs analyzed. They found that traffic and fossil fuel combustion contributed most to cancer risks in both cities, while industrial emissions were a major contributor to non-cancer risk in Incheon at 36.8%.

The aim of this work was to establish the share of different sources of air pollution to the total health hazard. The objectives include the design of the scheme, including choices regarding the processing of measurement data and the verification of the scheme with measurement and statistical data for Poland, including regional aspects. Obtaining this information for Poland was an additional objective.

The research question motivating this work was as follows. Can an emissions inventory, air pollution measurement database and the relative risk coefficients of air pollutants provide a useful estimation of the contributions of different sources of air pollution to the total health hazard?

The scheme proposed differs from earlier approaches in that the assessment is based solely on widely accessible public data: measurements from sensor networks, emission inventories and basic population data. Thus, the scheme can be easily used in any region or country in the world, where these data are available, and provides easy access to essential information for policymakers.

## 2. Methods

### 2.1. The Scheme and Its Test

The following general-purpose computational scheme is proposed:

(1) $$H_{i}=\sum_{k=1}^{N}c_{ik}\xi_{k},$$

where *H_i_*—health hazard from *i*-th source, *N*—the number of pollutants, *c_ik_*—the contribution of the *k*-th pollutant from source *i* in total emission of *k*-th pollutant, *ξ_k_*—the health hazard from *k*-th pollutant. This scheme is flexible; *c_ik_* will normally be expressed as a percentage, while *H_i_* and *ξ_k_* are expressed as an appropriate indicator of the user’s choice. The most comprehensible measures are attributable mortality or morbidity.

This is the general form, where the numbers of sources of pollution and pollutants can be set arbitrarily and *c_ik_* for each source is determined from source apportionment. The measure of *ξ* is flexible. In this work an estimation of the total number of premature deaths is attributable to a pollutant. This framework was applied to estimate the contributions from different sources to the total health hazard from air pollutants in Poland.

The hazards were expressed as all-cause mortality attributable to air pollutants, a frequently used indicator. The number of sources were limited to those most contributing to ambient air pollution, and the number of pollutants was limited to those with widely documented health effects. Two types of analyses were carried out: for long-term and short-term health effects.

The test is based on data from sensors and population data from all 16 provinces of Poland. This provided an intermediate result for health hazards from the main pollutants in all provinces of Poland. The emission inventory used was only available at the national level, so the final attribution of health hazards to sources of air pollution was determined nationally.

### 2.2. Pollutants

There is a group of air pollutants with the strongest evidence for public health concern. They are regulated by national standards and WHO guidelines. In the EU these are PM (both PM_2.5_ and PM_10_), O_3_ (ground level), nitrogen dioxide and sulfur dioxide. They are collectively referred to as key pollutants. In the U.S. two more substances are included: carbon monoxide and lead, and the group is called criteria pollutants.

In this work three pollutants were chosen as a basis for assessment of health hazards: PM_2.5_, O_3_ and NO_2_. There is little evidence for adverse health effects of the PM fraction between 2.5 and 10 μm; therefore, only PM_2.5_ was included and not PM_10_, although the latter has wider coverage in public networks of sensors. The three substances are commonly considered main air pollutants and have a big advantage when used to assess health effects as concentration–response functions (CRFs) based on numerous epidemiological studies have been determined for them [2,13,14].

In the analysis of short-term health hazards, the fourth pollutant was included: sulfur dioxide, since its short-term CRF has been determined recently [23].

### 2.3. Sensors

The public system of air quality monitoring in Poland is based on 291 measurement stations [4] distributed all over the country according to an appropriate directive of the European Union. Each station contains a couple of devices (sensors) measuring pollutants. The choice of sensors in each station is not fixed; some sensors are deployed more often than others. The total number of sensors is 1782, out of which 1022 are automatic, providing measurements every hour, and 760 are manual, providing measurements every 24 h. Automatic measurements are accessible online. In this analysis measurements from all sensors (both automatic and manual) of PM_2.5_ (about 150 sensors), all sensors of NO_2_ (about 150), all sensors of O_3_ (about 90) and all sensors of SO_2_ (about 85) were used.

Data published in yearly reports of the sensor network operator [4] were used. Before each yearly report is published, the operator provides filtering of outliers.

The sensors of gaseous pollutants conform to the following European standards: NO_2_—[24], based on chemiluminescence, O_3_—[25], based on photometry in UV, and SO_2_—[26], based on fluorescence in UV. The names of manufacturers are not provided to avoid commercialism, but are available from the authors.

The sensors of PM_2.5_ conform to the standard [27] (gravimetric method) or [28] (equivalent continuous measurement, based on optic principle or beta radiation). The measurement methods are reference ones and are used all over Europe, employing the same sensors with minor differences.

The monitoring system is operated by the Chief Inspectorate of Environmental Protection. The database of all past measurements is available at [4].

### 2.4. Sources and Source Apportionment

Following the objective of this study, the sources were grouped so that they share similar legal or administrative regulations, with one exception where properties of product or processes was a criterion for grouping (use of solvents). Similar groups can be found in many works on sources of air pollution, but the groups used in this paper followed those proposed in [10] most closely and their abbreviations were used (see Table 1).

The national emission inventory was used as crude estimation of source apportionment. It has a key advantage for the objective of this work—the data are readily available in countries or regions where inventories are held. In Poland, the yearly emission inventory is reported by the National Centre for Emissions Management [29]. Most of the emission estimates are based on methodologies described in EMEP/EEA Air Pollutant Emission Inventory Guidebook. Wherever necessary and possible, domestic methodologies have been developed.

Emissions in the inventory [29] are divided into several layers of subcategories. In this work, all appropriate subcategories were included to fit in one of the sources listed in Table 1, which contains the contributions thus obtained (in %) from sources to total emission of a given pollutant in Poland. Contributions below 1.5% of emission from one source were not included.

Uncertainties of data have been estimated in [29]. For the pollutants in question for this analysis, they amounted to 26.9% for PM_2.5_, 18.5% for NO_x_ (NO + NO_2_, NO_2_ is not inventoried separately), 12.1% for SO_2_ and 21.8% for non-methane volatile organic compounds (NMVOCs).

The inventory contains all key pollutants and some other pollutants of concern, except for O_3_, which is a secondary pollutant. Therefore, in this work O_3_ emissions were estimated indirectly, from emissions of O_3_ precursors: NMVOCs and NO_x_. Methane (formally a VOC) is routinely excluded from the analyses of air pollution, since (i) it is not harmful in usual concentrations in ambient air and (ii) it is not included in the analyses of O_3_ precursors, since it does not contribute as much to the formation of O_3_ ozone as other VOCs. When estimating emissions of O_3_ from its precursors, a problem arises of an appropriate proportion. While formation of O_3_ is most efficient at the mass ratio of VOCs to NO_x_ of 8:1 [30], the proportion of ambient concentrations is usually much lower, not considerably exceeding 1:1 [31]. The sensitivity of O_3_ formation to VOCs and NO_x_ is a complex issue [30,32,33] and at low values of VOC-to-NO_x_ ratio (VOC limited formation regime), it is positive for VOCs but tends to be negative for NO_x_. This would suggest the exclusion of NO_x_ from the estimation, when the final goal is the reduction in O_3_. Yet, there are works indicating that NO_x_ regulation reduces ozone levels over the long term, but can also be effective for reducing peak ozone concentrations [34]. Therefore, it was chosen to calculate the contributions of O_3_ precursor sources taking the full amount of NMVOCs inventoried and 1/8 of NO_x_ inventoried, according to

(2) $$C_{i}=\frac{E_{NMVOC\left(i\right)}+{0.125E}_{NOx\left(i\right)}}{E_{NMVOC}+0.125E_{NOx}}$$

where *C_i_*—the contribution of source *i* in total emissions of O_3_ precursors (NMVOC and NO_x_) [%], *E*_NMVOC(*i*)_—emissions of NMVOC from source *i* [kilotons/year, kt/y], *E*_NOx(*i*)_—emissions of NMVOC from source *I* [kt/y], *E*_NMVOC_—total emissions of NMVOC [kt/y], *E*_NOx_—total emissions of NO_x_ [kt/y].

The inventory specifies emissions of NO_x_ while a pollutant of concern is NO_2_. NO_2_/NO_x_ ratios at particular sources of emission are not an appropriate indicator of emission, due to processes in the atmosphere. The NO_2_/NO_x_ ratio in the ambient air ranges from 0.3 to 0.6 [35,36] (0.25 to 0.35 at traffic sites). Therefore, proportions of emissions of NO_x_ among particular sources can be used as a substitute measure of contributions of NO_2_ from these sources in ambient air.

### 2.5. Estimation of Health Hazards

Health hazards are often expressed as a rate of premature deaths attributable to a particular air pollutant. This approach was also used in this work. The relative risk (*RR*) of a premature death is determined based on CRFs. This coefficient is subsequently used in the procedure for the estimation of mortality attributable to a particular air pollutant. The commonly used WHO methodology has recently been updated [37].

Out of those specified in [37], the following numbers were chosen as counterfactual values of concentration: 5 μg/m^3^ for PM_2.5_, 10 μg/m^3^ for NO_2_ (the baseline values according to Soares et al. [37]) and 60 μg/m^3^ for O_3_ (baseline value adjusted). The rationale for the adjustment of the last value was that the baseline value of counterfactual concentration at 70 μg/m^3^ seems overestimated in view of the current guideline value set by the WHO at 60 μg/m^3^.

The most frequently used *RRs* are those obtained from epidemiological studies on all-cause mortality from long-term exposures. These coefficients have higher certainty than *RRs* for short-term exposures [1] or those for morbidity endpoints [38]. Epidemiological studies on mortality due to long-term exposure to SO_2_ have been carried out, but the variability of *RR* coefficients found was high [39]; therefore, health risks from long-term exposure to SO_2_ was not included in the present analysis. The short-term *RR* for SO_2_ demonstrates a substantially narrower confidence interval, so short-term SO_2_ exposure was included. The list of *RR* values established by WHO and counterfactual values of concentration used is given in Table 2.

The exposures were calculated in the following way. For the long-term PM_2.5_ and NO_2_ exposures, the yearly average values of concentrations (all monitoring sites) were applied. For O_3_ it was peak-season (from April through September) average of 8 h daily maxima. For the short-term exposures to PM_2.5_, NO_2_ and SO_2,_ the lowest value of the four highest 24 h averages in a year (99th percentile) were used, while for O_3_, it was the lowest value of four 8 h highest averages in a year. The data from both automatic and manual sensors were included in the averages. This improved accuracy, since some differences occur even when both types of sensors are installed in the same measuring location. In the case of O_3_ only measurements from automatic sensors were used, as 8 h daily maxima are included in the averages.

When estimating health hazards from a number of sources according to (1), hazards from different pollutants should be summed. However, they are non-additive. Integrating impacts of multiple pollutants is complicated, because possible interactions between them may bring antagonistic or synergistic effects [16,40]. This is further complicated by the collinearity of pollutants. Despite research effort, the results are still divergent or inconclusive. While some two-pollutant and multipollutant models indicate that pollutants mutually mitigate their effects, especially when PM is present in the mixture [41,42], other works suggest that considerable synergistic effects may occur, especially when O_3_ is present in the mixture [43,44,45,46].

The following approach was adopted. For PM_2.5_ interaction with O_3_, and for NO_2_ interaction with O_3_, no grounds were found to apply adjustments either way, i.e., for antagonistic or synergistic effects, and straight summation of single-pollutant effects was used. In the case of PM_2.5_ and NO_2_ interaction, it was chosen to use the results of a systematic review of cohort studies in Chen et al. [42]. An important statistical parameter is referred to as population attributable fraction (*PAF*) and is defined as the proportional reduction in population disease or mortality that would occur if exposure to a risk factor were reduced to an alternative ideal exposure scenario. One of the main results in [42] is that average *PAF* when calculated from the sum of PM_2.5_ and NO_2_ single-pollutant models amounts to 0.079, while the average *PAF* calculated from two-pollutant models for PM_2.5_ and NO_2_ is 0.057. This gives a simple adjustment coefficient for the *PAF* of 0.722, which can be applied at the final stage of the analysis. The adjustment for *PAF* cited has been determined at standardized increments of pollutants: 5 μg/m^3^ for PM_2.5_ and 10 μg/m^3^ for NO_2_. This involves an important assumption; the ratio of NO_2_ to PM_2.5_ in ambient air amounts to two. The ratio of long-term averages for these two pollutants in Poland is lower, i.e., below one. In [42] they also specified the effects of interaction on *RRs*, which leads to the conclusion that a mitigating effect of NO_2_ on PM_2.5_ is stronger than that of PM_2.5_ on NO_2_. Therefore, it was assumed that the mitigating effect of NO_2_ on PM_2.5_ decreases linearly from the value determined at the ratio of NO_2_ to PM_2.5_ of two, hypothesizing zero mitigation at zero concentration of NO_2._ Hence, regional *PAF*s in this analysis were calculated according to

(3) $${PAF}_{ra}=\left(1-\left(1-0.722\right)\frac{\frac{C_{NO2r}}{C_{PM2.5r}}}{2}\right){PAF}_{r},$$

where *PAF_ra_*—regional *PAF*, adjusted according to two-pollutant NO_2_ and PM_2.5_ model, *C*_NO2*r*_—long-term regional concentration of NO_2_ (or PM_2.5_ accordingly) [μg/m^3^], *PAF_r_*—regional *PAF* unadjusted.

### 2.6. Population Data and Analysis of All-Cause Mortality

An analysis of all-cause mortality attributed to the main air pollutants in all provinces of Poland was performed (provinces corresponded to regions in (3)). Statistics on population and mortality were extracted from the database of Statistics Poland agency [47].

At the time of writing this paper, verified data from sensors and population data were available for the year 2024, but the most recent emission inventory contained data for 2023. Therefore, all data used in this analysis refer to 2023.

In the analysis of all-cause mortality, the updated WHO methodology [37] was followed. The procedure is summarized below with the symbols adjusted to the approach used in this work. In the first step, the *RR* of all-cause death attributable to a pollutant is calculated:

(4) $${RR}_{pC}=exp\left[\beta \left(C-C_{0}\right)\right],$$

where *RR_pC_* is a relative risk from pollutant *p* at average concentration *C* [μg/m^3^], *β* is *RR*-1 (*RR* as in Table 2), *C* is average concentration in tens of μg/m^3^, *C*_0_ is counterfactual concentration. Then *PAF_pC_* (*PAF* from pollutant *p* at average concentration *C*) is determined according to

(5) $${PAF}_{pC}=\frac{{RR}_{pC}-1}{{RR}_{pC}}.$$

Finally, the number of premature deaths in a given area (province) is calculated from

(6) $$D_{pC}={PAF}_{pC}\cdot M\cdot P,$$

where *D_pC_* is the number of deaths attributable to pollutant *p* at average concentration *C*, *M* is the mortality rate [deaths/1000 inhabitants] and *P* is the population of a province.

The data from [4] were imported into statistical package R (version 4.4.1), where the main computations were performed. Some calculations were performed in Matlab (version 2025a).

### 2.7. Summary of Steps

1. Acquisition of all data: a complete database of measurements of PM2_2.5_, NO_2_, O_3_, and SO_2_ from the national sensor network; a national inventory database for Poland; demographic data for Poland by province.
2. Processing of data on pollutants to obtain appropriate estimators of average concentrations used in (3) and (4).
3. Computing estimated numbers of premature deaths by province, according to (4) to (6), presented in Table 3 and Table 4, and at the national scale, presented in Table 5. In these computations, (i) the data on concentrations from [4] were used (specifically, the files for the year 2023: NO_2_ 1 h and 24 h measurements, O_3_ 1 h, PM_2.5_ 1 h and 24 h, SO_2_ 1 h and 24 h) and (ii) the population and mortality data for each province (voivodeship) from [47] (Table 1).
4. Computing estimated numbers of premature deaths from air pollution attributable to its main sources, according to (1), with the numbers from Table 5 used as *ξ_k_*, and the values in Table 1 as *c_ik_.*

## 3. Results

### 3.1. Mortality Analysis by Province

Table 3 and Table 4 contain the estimated numbers of premature deaths (calculated according to (4) to (6)) per 100,000 inhabitants, attributed to main air pollutants in Poland by province, from long-term and short-term exposures, respectively.

### 3.2. Attribution of Health Hazards to Sources at the National Level

While the numbers in Section 3.1 were obtained for each province, the attribution of health hazards to sources was computed at the national level. The total numbers of premature deaths attributable to each of the main pollutants in Poland in all provinces were calculated. The results are given in Table 5.

**Figure 1:**
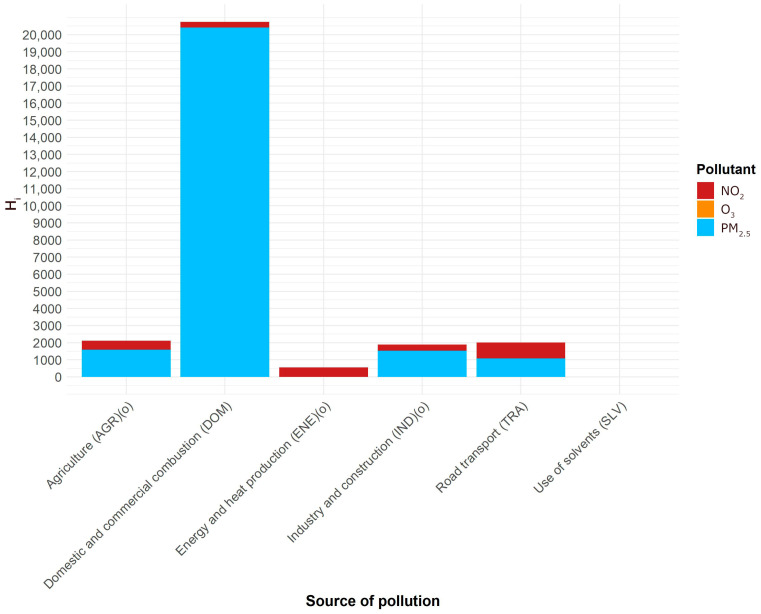
The number of premature deaths attributable to the main sources of air pollution in Poland, estimated for the year 2023. The composition of emissions is shown in colors. Long-term exposure. (o): overestimated.

**Figure 2:**
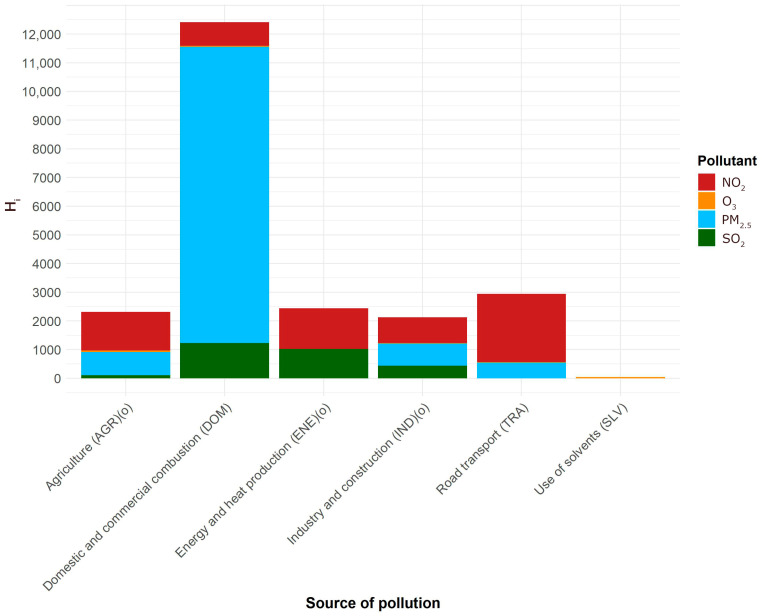
The number of premature deaths attributable to the main sources of air pollution in Poland, estimated for the year 2023. The composition of emissions is shown in colors. Short-term exposure. (o): overestimated.

The numbers from Table 5 were used in (1) as *ξ_k_*, and the values in Table 1 as *c_ik_.* The numbers of premature deaths from air pollution attributable to its main sources are presented in Figure 1 and Figure 2.

In both cases of long- and short-term effects, by far the largest source of health hazard is domestic and commercial combustion. This conforms to a widely accepted conviction, based on source apportionment, but so far no quantitative assessment was available of the health hazard it creates. The health hazards from long- and short-term exposures should not be added, as short-term exposures are included in long-term exposures. The attributions of deaths to sources are not expressed as percentages as in principle they are not additive.

## 4. Discussion

While source apportionment of air pollutants is a well investigated area, the attribution of health hazards to sources has hardly been attempted. Kim et al. [22] prioritized sources of HAPs according to their health effects in a big city environment in Korea. They have attained their goal, providing a list of sources with corresponding quantitative measures of their cancer and non-cancer risks and thus obtained a clear ranking of their harmfulness. They expressed these measures in percentages of the total health risks from 60 HAPs investigated. This method of presentation is very useful to policy makers, albeit the relative contributions of different pollutants are in general non-additive.

The work of Kim et al. [22] is useful as a reference and pattern to follow, but obtaining similar information relevant to any other location requires considerable workload. Concentrations of HAPs are very location-specific, so their results are hardly applicable elsewhere. Furthermore, HAPs are pollutants of concern in highly industrialized areas, but it is widely accepted that key pollutants bring more health hazards in all other areas.

The aim of this work was formally similar to theirs, but here it was approached with very different assumptions: (i) to achieve an easy-to-apply scheme of calculations, to be fed only with accessible data and (ii) to estimate health hazards from the pollutants widely accepted as most harmful in the scale of world population, that is, PM_2.5_, NO_2_ and O_3_. A further advantage of this choice of pollutants is that their health effects are based on numerous epidemiologic studies with large cohorts, and hence have low uncertainty, unlike HAPs, where risk coefficients are mostly obtained by extrapolation to humans from laboratory research.

The accuracy of the estimation of exposure to pollutants in the proposed scheme was adequate thanks to the reference quality of sensors used in the public network and analyses of exposure in different provinces of the country, which made the results closer to population-weighted exposure.

The main limitation of the presented scheme is the uncertainty of source apportionment of pollutants based on emission inventory. No model of dispersion and transformation processes in the atmosphere between the source and the receptor (sensor) was used. This increased uncertainties of the results, but three of the sources listed in Table 1—DOM, TRA and SLV—can be considered local, close to the receptor (a man or sensor). Both transport and heating are very close to receptors. The SLV group of sources, responsible for the formation of O_3_, are partly close to human dwellings and partly far away, but distant sources do not contribute as O_3_ is a short-lived gas. The above qualitative statement is supported (at least in the case of PM_2.5_) by the finding reported in [21] that local emissions contribute more to PM_2.5_-related deaths than PM_2.5_ concentration. With this approach, estimations of contributions from DOM, TRA and SLV seem reasonable, but the assessments for AGR, ENE and IND are overestimated, since sources are usually more distant from the receptors. Since quantifying the overestimation was not attempted, in the final estimation the values for these sources were denoted “(o)”.

Although source apportionment seems a large source of uncertainty in the proposed scheme, results of source apportionment of PM_10_ carried out with an advanced method involving chemical analysis for the content of water-soluble ions, carbonaceous matter and trace elements [48] averaged for two locations in southern Poland provided results close to those compiled in this work on the basis of the national emissions inventory. Residential coal and wood combustion were the source of 47% of PM_10_ (consisting around 70% of PM_2.5_), road transport of 17.5%. Furthermore, 88% of PM_10_ originated from local sources, as was hypothesized above.

On the other hand, uncertainty in the assessment of health risks is at the lowest possible level, due to relatively low confidence intervals of *RR* coefficients [37]. The assessments of risk factors of HAPs may differ by an order of magnitude or more [22]. The *RR* coefficients used in this work are reliable in one-pollutant models, but according to the construction of the scheme in (1), an additive three-pollutant model was used, while interactions were unknown. This effect was mitigated by adjusting the results according to the findings in [42]. The other mitigating effect comes from the fact that the frequently mentioned correlation between the pollutants is limited. Even for the pair with widely accepted correlation (PM_2.5_ and NO_2_), it is less pronounced in shorter time scales and varies locally [49]. Furthermore, there is an agreement that the correlation between NO_2_ and O_3_ is negative. The conclusion is that it is not common for two or three main pollutants to be simultaneously present at proportions enhancing interactions.

Although *RR*s based on epidemiological studies with large cohorts of subjects provide the best estimation of health hazards, their use in countries outside North America and Western Europe may introduce bias, as most epidemiological studies were carried out there. Elsewhere, the pollution levels, chemical composition of the PM and ground-level atmosphere and also healthcare systems may be different.

The scheme proposed was tested with characteristics for one year. A longer period, e.g., three years, might provide more reliable estimates that are less dependent on meteorological conditions, but emissions in Poland have fallen fast in recent years, so one year was chosen to provide more recent estimates.

## Figures and Tables

**Table 1:** Sources of air pollution with strongest health effects in Poland used in the further analysis and their contributions to total emission of each pollutant, in %. Columns do not sum to 100% as minor sources are not included in the inventory.

			Pollutant		
Source of Pollution	PM_2.5_	NO_2_	O_3_ *	SO_2_ **	NMVOC ***
Agriculture (AGR)	5.9	18	28	3.8	29
Domestic and commercial combustion (DOM)	76	11	17	42	18
Energy and heat production (ENE)		19	1.6	35	
Industry and construction (IND)	5.7	12	7.7	15	7.3
Use of solvents (SLV)			21		23
Road transport (TRA)	4	32	9		6.7

* Estimated from emission of precursors. ** SO_2_ data were only used for short-term analysis. *** Used only for the estimation of precursors to O_3._

**Table 2:** Main pollutants, their relative risks (*RRs*) and counterfactual concentrations (from [1,23,37]).

Pollutant	Long-Term *RR*	Short-Term *RR*	Counterfactual Concentration, C_0_ [μg/m^3^]
PM_2.5_	1.08	1.0065	5
NO_2_	1.02	1.0072	10
O_3_	1.01	1.0043	60
SO_2_	-	1.0059	0

**Table 3:** The number of premature deaths per 100,000 inhabitants by province, attributable to main air pollutants in long-term exposure.

	The Number of Premature Deaths Per 100,000, Attributable To:		
Province	PM_2.5_	NO_2_	O_3_
Dolnośląskie	67.8	6.0	0.0
Kujawsko-pomorskie	63.8	6.7	0.0
Lubelskie	77.1	0.0	0.0
Lubuskie	54.7	0.0	0.0
Łódzkie	98.9	10.7	0.0
Małopolskie	68.8	14.8	0.0
Mazowieckie	68.9	10.9	0.0
Opolskie	75.0	4.5	0.0
Podkarpackie	57.7	1.9	0.0
Podlaskie	54.0	1.2	0.0
Pomorskie	44.5	1.5	0.0
Śląskie	93.7	17.6	0.0
Świętokrzyskie	80.6	14.4	0.0
Warmińsko-mazurskie	49.7	0.0	0.0
Wielkopolskie	83.3	3.6	0.0
Zachodniopomorskie	57.0	4.2	0.0

**Table 4:** The number of premature deaths per 100,000 inhabitants by province, attributable to main air pollutants in short-term exposure.

	The Number of Premature Deaths Per 100,000, Attributable To:			
Province	PM_2.5_	NO_2_	O_3_	SO_2_
Dolnośląskie	41.9	19.9	0.0	8.0
Kujawsko-pomorskie	36.2	19.0	0.0	4.5
Lubelskie	34.8	14.7	0.0	4.3
Lubuskie	32.8	11.9	0.0	8.8
Łódzkie	51.2	22.7	0.0	9.3
Małopolskie	43.3	23.4	0.0	8.1
Mazowieckie	27.8	20.9	0.2	6.6
Opolskie	29.7	19.4	0.0	7.3
Podkarpackie	31.7	11.9	4.1	4.5
Podlaskie	21.2	10.5	0.0	2.5
Pomorskie	23.1	13.4	0.0	3.9
Śląskie	40.8	30.9	0.0	16.3
Świętokrzyskie	39.7	34.3	0.0	9.2
Warmińsko-mazurskie	22.5	9.7	0.0	7.9
Wielkopolskie	45.7	16.8	0.0	6.5
Zachodniopomorskie	34.7	17.0	6.7	8.2

**Table 5:** Numbers of premature deaths attributable to each of the main pollutants in Poland in 2023, long- and short-term effects.

	PM_2.5_	NO_2_	O_3_
Long-term effects	26,865.3	2928.6	0.0
Short-term effects	13,589.7	7451.5	203.8

## Data Availability

The original contributions presented in this study are included in the article. Further inquiries can be directed to the corresponding author.

## Abbreviations

The following abbreviations are used in this manuscript:

WHO	World Health Organization
UNECE	United Nations Economic Commission for Europe
EPA	Environmental Protection Agency
ROW	Rest of the World
HAP	Hazardous air pollutant
CRF	Concentration-response function
EMEP	European Monitoring and Evaluation Programme
EEA	European Environment Agency
VOC	volatile organic compounds
NMVOC	Non-methane volatile organic compounds
AGR	Agriculture
DOM	Domestic and commercial combustion
ENE	Energy and heat production
IND	Industry and construction
SLV	Use of solvents
TRA	Road transport
NO_x_	NO and NO_2_ measured together
*RR*	Relative risk (of premature death)
*PAF*	Population attributable fraction
*H*_i_	Health hazard from *i*-th source
*c_ik_*	Contribution of the *k*-th pollutant from source *i* in total immission of *k*-th pollutant
*ξ_k_*	Health hazard from *k*-th pollutant
*C*	Average concentration of a pollutant
*C*_0_	Counterfactual concentration of a pollutant
